# Construction of risk prediction model for hyponatremia in patients with acute decompensated heart failure

**DOI:** 10.1186/s12872-023-03557-5

**Published:** 2023-10-26

**Authors:** Huanhuan Gong, Ying Zhou, Yating Huang, Shengen Liao, Qin Wang

**Affiliations:** The First Affiliated Hospital of Nanjing Medical University, Nanjing, China

**Keywords:** Acute decompensated heart failure, Hyponatremia, Risk prediction

## Abstract

**Background:**

Patients with Heart failure (HF) commonly have a water-electrolyte imbalance due to various reasons and mechanisms, and hyponatremia is one of the most common types. However, currently, there are very few local studies on hyponatremia risk assessment in patients with acute decompensated heart failure (ADHF), and there is a lack of specific screening tools. The aim of this study is to identify a prediction model of hyponatremia in patients with acute decompensated heart failure (ADHF) and verify the prediction effect of the model.

**Methods:**

A total of 532 patients with ADHF were enrolled from March 2014 to December 2019. Univariate and multivariate logistic regression analyses were performed to investigate the independently associated risk factors of hyponatremia in patients with ADHF. The prediction model of hyponatremia in patients with ADHF was constructed by R software, and validation of the model was performed using the area under the receiver operating characteristic curve (AUC) and calibration curves.

**Results:**

A total of 65 patients (12.2%) had hyponatremia in patients with ADHF. Multivariate logistic regression analysis demonstrated that NYHA cardiac function classification (NYHA III vs II, OR = 12.31, NYHA IV vs II, OR = 11.55), systolic blood pressure (OR = 0.978), serum urea nitrogen (OR = 1.046) and creatinine (OR = 1.006) were five independent prognostic factors for hyponatremia in patients with ADHF. The AUC was 0.757; The calibration curve was near the ideal curve, which showed that the model can accurately predict the occurrence of hyponatremia in patients with ADHF.

**Conclusions:**

The prediction model constructed in our study has good discrimination and accuracy and can be used to predict the occurrence of hyponatremia in patients with ADHF.

## Introduction

Heart failure (HF) is a clinical syndrome characterised by cardiomyocyte injury and structural and functional changes due to various reasons, which ultimately leads to ventricular blood pumping and (or) filling decline, thus failing to meet the body’s needs. Acute decompensated heart failure (ADHF) has emerged as a global public health problem owing to its high incidence rate, rehospitalisation rate and mortality [1–3]. Patients with HF commonly have a water-electrolyte imbalance due to various reasons and mechanisms, and hyponatremia is one of the most common types [4–6]. According to earlier reports [7–11], the hyponatremia incidence ranged between 7.2%–27% in inpatients with HF. Meanwhile, hyponatremia is independently associated with poor HF patient prognosis [12]. The incidence of adverse events also increases along with the increase in the degree of hyponatremia [13–15]. Therefore, it is of great significance to maintain blood sodium balance in patients with HF. There are presently a few local studies on hyponatremia risk assessment in ADHF patients. There is a lack of specific screening tools.

A nomogram is a widely used prognostic model that can intuitively reflect the predictive ability of prognostic factors based on the length of line segments. A nomogram can guide clinicians to make rapid and comprehensive judgments and help them make individualized clinical decisions [16]. We have performed a retrospective analysis of the patients with ADHF in a third-grade hospital. The large sample size of the hospital facilitated reliable research conclusions.

The present study was carried out to construct a risk prediction model by analysing the influential factors of hyponatremia in patients with ADHF. Our study is expected to provide nursing staff with specific screening tools for hyponatremia risk in patients with ADHF.

## Materials and methods

### Study population

The study participants were 590 patients who were admitted to a third-grade hospital for ADHF from March 2014 to December 2019. This study was approved by the ethics committee.

**Fig. 1:**
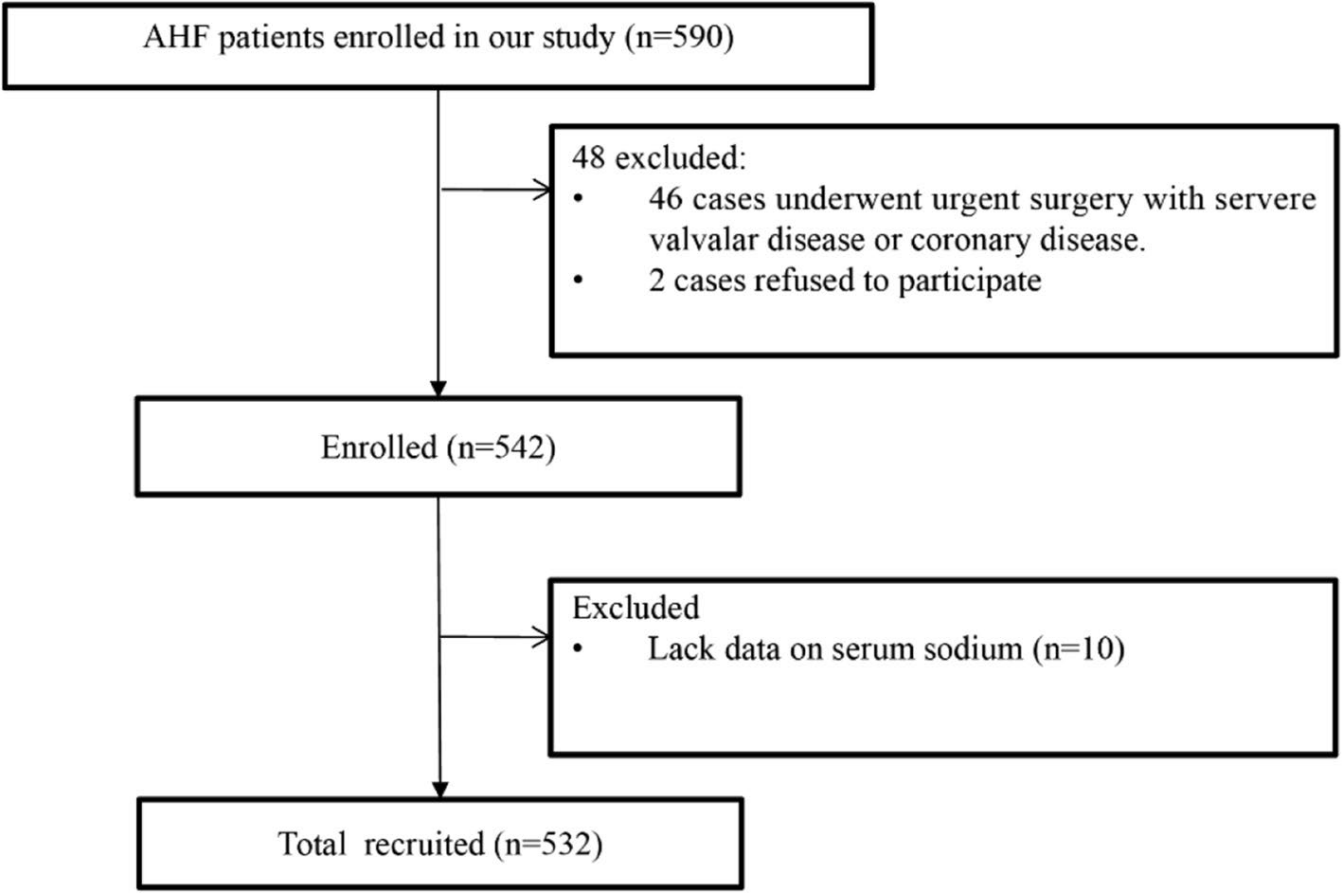
Study flow chart

Inclusion criteria: patients with ADHF according to the definition in the Guidelines for diagnosis and treatment of HF [17]. Exclusion criteria: patients with malignancies, patients with cognitive impairment and dementia, patients with severe mental illness, patients with primary hepatic and renal failure and patients with other severe uncontrollable systemic diseases. Finally, we included 532 patients (Fig. 1).

### Definition of hyponatremia

The enrolled patients were divided into the normal blood sodium group (serum sodium ≥ 135 mmol/L) and the hyponatremia group (serum sodium < 135 mmol/L) [11] according to the results of the serum sodium test on the day of admission.

### Covariates

The baseline data and serum samples were collected from the enrolled patients within 24 h after admission, associated with echocardiography completion and other instrumental examinations within 48 h. The following data were collected, including: (1) demographic data: name, age, gender, height and weight; (2) disease information: primary aetiology and comorbidities of HF (diabetes, hypertension, coronary heart disease, etc.); (3) laboratory examinations: albumin, creatinine, serum urea nitrogen, C-reactive protein, N-terminal pro-brain natriuretic peptide (NT-proBNP), etc.; and (4) instrumental examination: echocardiography.

### Statistical analysis

SPSS 22.0 and R 3.6.0 were used for statistical analysis. Qualitative data were expressed in (%), and the chi-square test was used for inter-group comparison. Meanwhile, quantitative data were presented in the form of mean ± standard deviation (x ± s) or median (interquartile range), which were compared using an independent sample t-test or rank sum test between groups. Univariate logistic regression analysis was performed to analyse the potentially related hyponatremia factors. Multivariate logistic stepwise regression analysis was conducted to screen the independent related hyponatremia factor, during which indicators with *P* < 0.05 in univariate analysis were included in the model, and indicators with *P* > 0.05 in multivariate analysis were excluded from the model. Nomogram model drawing and internal validation adopted the R 3.6.0 software package. The receiver operating characteristic (ROC) curve was additionally drawn, and the area under the ROC curve (AUC) was calculated to identify the accuracy of the model. The calibration curve was applied to evaluate the consistency between the predicted value and the actual value of the prediction model. *P* < 0.05 meant that the difference was statistically significant.

## Results

### Baseline characteristics

Of the 532 patients enrolled in this study, there were 353 males (66.4%), and the average age was (61.0 ± 16.0) years old. Patients with New York Heart Association (NYHA) cardiac function grades II, III and IV accounted for 16.9%, 53.4% and 29.7% of the total cases, respectively. The average EF value was 42.3% ± 14.6%. Meanwhile, 65 patients (12.22%) were identified to have hyponatremia (serum sodium < 135 mmol/L). Table 1 shows the baseline data of patients grouped based on hyponatremia existence. It was found that there were statistically significant differences between groups in NYHA cardiac function grades, blood pressure, serum potassium, albumin, creatinine, uric acid, urea nitrogen, glutamic oxaloacetic transaminase, NT-proBNP and body mass index (all *P* < 0.05).

**Table 1:** Baseline characteristics

Variables	Total (*n* = 532)	Normal blood sodium (*n* = 467)	Hyponatremia (*n* = 65)	*P* value
Age (year)	61.0 (16.0)	60.8 (16.0)	62.8 (16.3)	0.362
Male, n(%)	353 (66.4%)	315 (67.5%)	38 (58.5%)	0.195
Diabetes mellitus, n(%)	131 (24.6%)	112 (24.0%)	19 (29.2%)	0.443
Hypertension, n(%)	272 (51.1%)	244 (52.2%)	28 (43.1%)	0.210
Ischemic heart failure, n(%)	137 (25.8%)	119 (25.5%)	18 (27.7%)	0.818
NYHA class				**0.002**
II	90 (16.9%)	89 (19.1%)	1 (1.5%)	
III	284 (53.4%)	245 (52.5%)	39 (60.0%)	
IV	158 (29.7%)	133 (28.5%)	25 (38.5%)	
Heart rate (beats/min)	85.3 (21.2)	85.1 (21.0)	86.7 (23.1)	0.450
Systolic blood pressure (mmHg)	127 (22.2)	128 (21.4)	119 (26.0)	**< 0.001**
Diastolic blood pressure (mmHg)	78.4 (15.0)	78.9 (14.5)	74.7 (17.9)	**0.002**
Potassium (mmol/L)	3.99 (0.50)	3.97 (0.48)	4.16 (0.63)	**0.008**
Albumin (g/L)	36.8 (4.91)	37.0 (4.62)	35.7 (6.57)	**0.014**
Creatinine (umol/L)	101 (55.2)	98.0 (49.1)	125 (83.9)	**0.011**
Uric acid (umol/L)	490 (170)	478 (161)	577 (204)	**< 0.001**
Urea nitrogen (mmol/L)	8.75 (5.68)	8.31 (5.34)	11.9 (6.96)	**< 0.001**
Alanine aminotransferase (U/L)	25.95(16.5,44.02)	25.7 (16.5, 42.75)	33.2 (18.0, 55.1)	0.146
Aspartate aminotransferase (U/L)	27.7 (21.7, 41.75)	27.1 (21.3, 39.4)	37.4 (26, 54)	**< 0.001**
pro B-type natriuretic protein (ng/L)	2231 (1269, 5847)	2124 (1232.5, 5475.5)	2893 (1728, 7818)	**0.002**
Left ventricular ejection fraction (%)	42.3 (14.6)	42.4 (14.7)	41.2 (14.0)	0.615
Body mass index (kg/M^2^)	24.1 (5.63)	24.3 (5.50)	22.6 (6.34)	**0.025**
Antisterone, n(%)	473 (88.9%)	416 (89.1%)	57 (87.7%)	0.902
ACEI/ARB, n(%)	412 (77.4%)	358 (76.7%)	54 (83.1%)	0.317
β blocker, n(%)	423 (79.5%)	372 (79.7%)	51 (78.5%)	0.952
Aspirin, n(%)	223 (41.9%)	191 (40.9%)	32 (49.2%)	0.254
Diuretic, n(%)	508 (95.5%)	445(95.1%)	63(95.4%)	0.226
Loop diuretic, n(%)	503 (94.7%)	441(94.2%)	62 (98.4%)	0.163
Serum sodium(mmol/L)	139.6 (3.9)	140.6(2.8)	132.2(2.8)	< 0.001

*Abbreviations*: *NYHA* New York Heart Association, *ACEI* angiotensin converting enzyme inhibitors, *ARB* angiotensin receptor blocker

### Analysis of related factors of hyponatremia

The indicators with statistically significant differences in univariate analysis were included in multivariate stepwise logistic regression analysis. As shown in Table 2, NYHA cardiac function classification (NYHA III vs II, OR = 12.31, NYHA IV vs II, OR = 11.55), systolic blood pressure (OR = 0.978), serum urea nitrogen (OR = 1.046) and creatinine (OR = 1.006) were the independent prognostic factors for hyponatremia in patients with ADHF (all *P* < 0.05).

**Table 2:** Univariate and multivariate analysis of the factors associated with hyponatremia

Variables	Univariate analysis		Multivariate analysis	
	OR(95%CI)	*P* value	OR(95%CI)	*P* value
Age	1.008(0.991, 1.025)	0.358		
Male	0.679(0.400, 1.154)	0.152		
Diabetes mellitus	1.309(0.737, 2.327)	0.359		
Hypertension	0.692(0.410, 1.167)	0.167		
Ischemic heart failure	1.08(0.80, 1.46)	0.614		
NYHA class				
II	**1.00**		**1.00**	
III	**14.17(1.92, 104.65)**	**0.009**	**12.31(1.66, 91.44)**	**0.014**
IV	**16.73(2.23, 125.70)**	**0.006**	**11.55(1.51, 88.14)**	**0.018**
Heart rate	1.004(0.992, 1.016)	0.551		
Systolic blood pressure	**0.981(0.968, 0.994)**	**0.005**	**0.978(0.965, 0.992)**	**0.002**
Diastolic blood pressure	**0.979(0.960, 0.999)**	**0.035**		
Potassium	**2.102(1.260, 3.507)**	**0.004**		
Albumin	**1.003(1.002, 1.005)**	**< 0.001**		
Creatinine	**1.085(1.041, 1.132)**	**< 0.001**	**1.006(1.001, 1.011)**	**0.024**
Uric acid	**1.006(1.002, 1.010)**	**< 0.001**		
Urea nitrogen	0.951(0.902, 1.002)	0.060	**1.046(1.004, 1.090)**	**0.034**
Alanine aminotransferase	1.001(1.000, 1.002)	0.104		
Aspartate aminotransferase	**1.001(1.000, 1.002)**	**0.016**		
pro B-type natriuretic protein	**2.654(1.418, 4.965)**	**0.002**		
Left ventricular ejection fraction	0.994(0.976, 1.012)	0.522		
Body mass index	**0.948(0.906, 0.991)**	**0.019**		
antisterone	0.873(0.394, 1.934)	0.739		
ACEI/ARB	1.495(0.755, 2.959)	0.249		
β blocker	0.930(0.494, 1.752)	0.823		
Aspirin, n(%)	1.401(0.833, 2.357)	0.204		

pro B-type natriuretic protein was log10 transformed

### Construction of Nomogram prediction model of hyponatremia in patients with ADHF

**Fig. 2:**
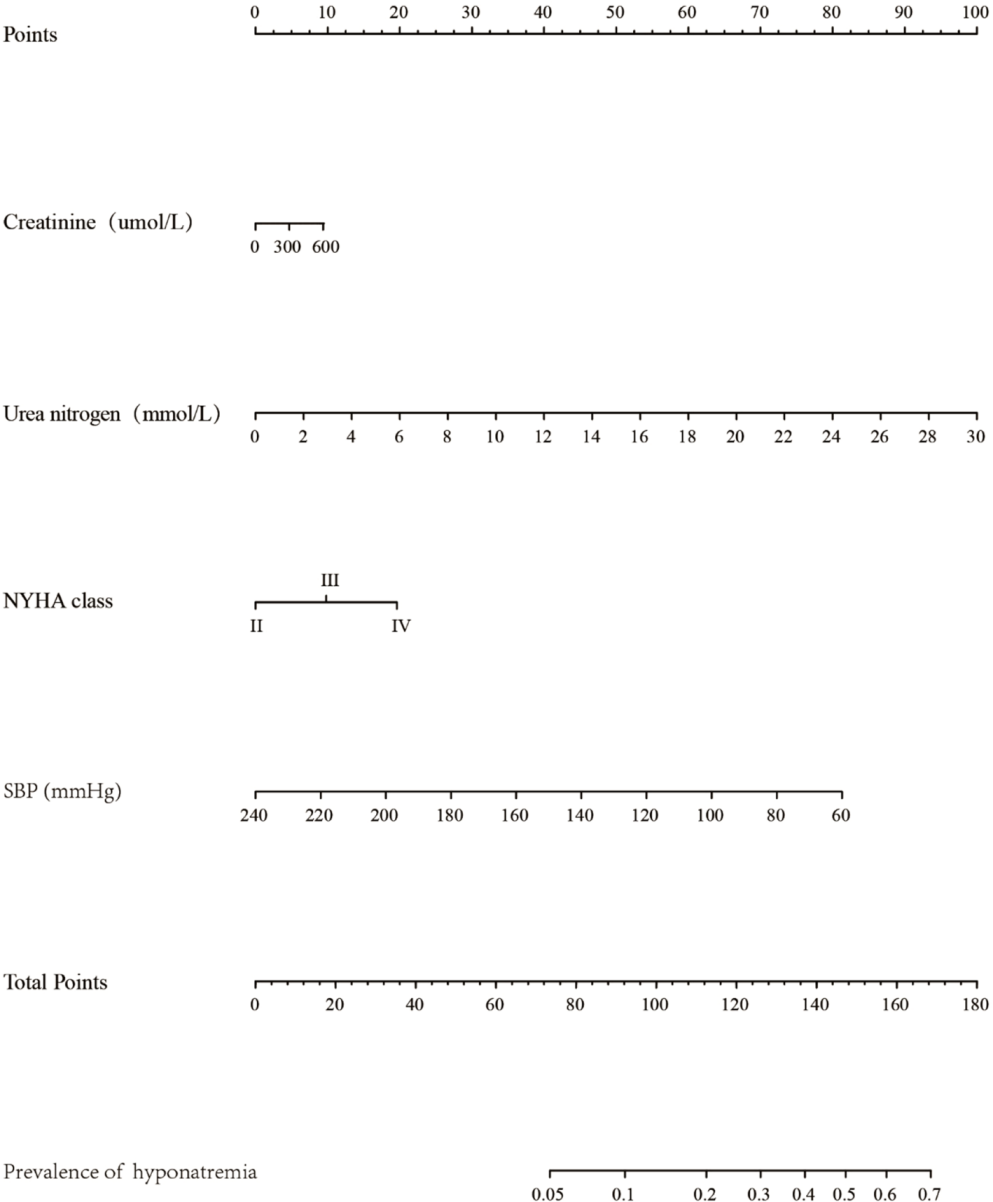
Predictive model for the development of hyponatremia in ADHF patients

The construction of the Nomogram model included the NYHA cardiac function classification, systolic blood pressure, urea nitrogen and creatinine to predict hyponatremia risk in ADHF patients, as presented in Fig. 2.

### Internal validation of Nomogram for predicting hyponatremia in patients with ADHF

**Fig. 3:**
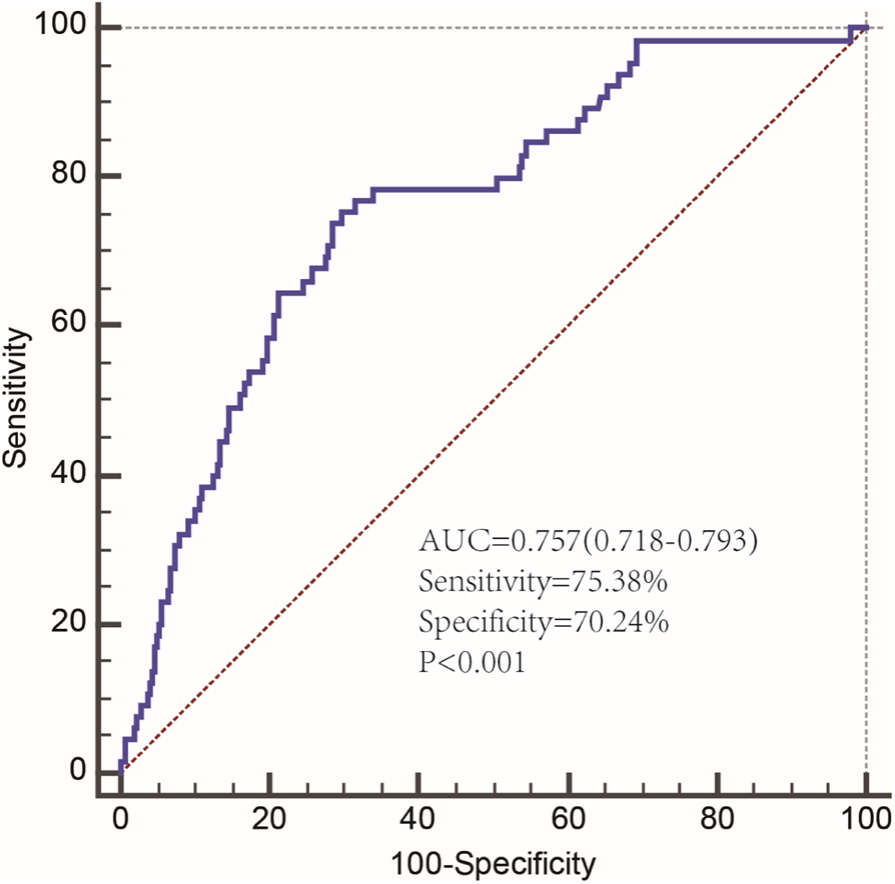
ROC curves of the prediction model

**Fig. 4:**
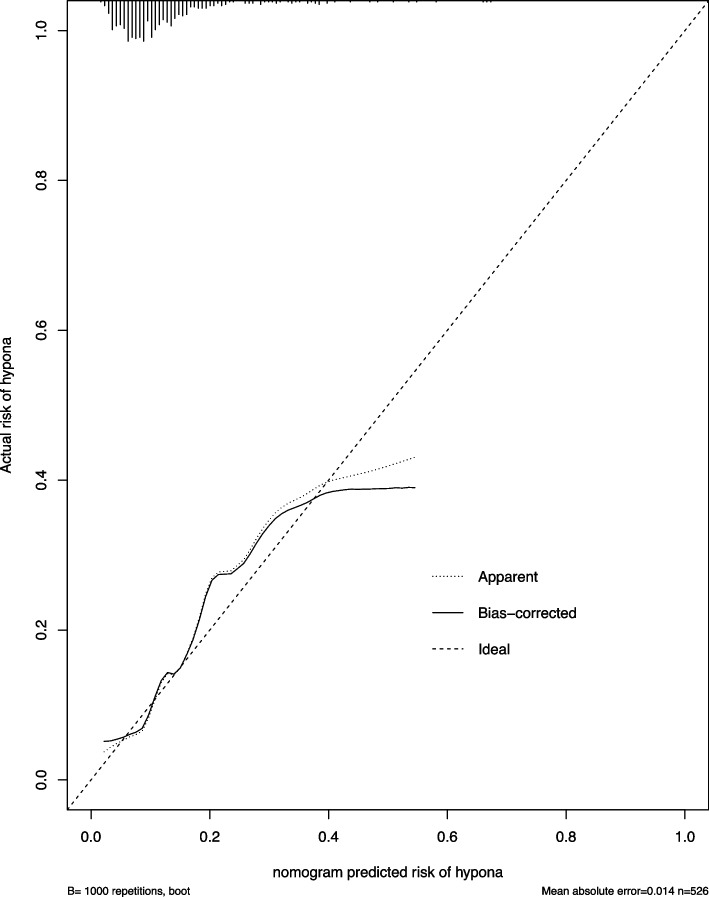
Calibration diagram

The AUC of the ROC was used to assess the accuracy of the constructed Nomogram model. The AUC was 0.757 (95% CI [0.718, 0.793], *P* < 0.001), suggesting that this model could predict hyponatremia occurrence. Meanwhile, the calibration curve revealed that the Nomogram model had good consistency in predicting hyponatremia occurrence in ADHF patients. The results are shown in Figs. 3 and 4.

## Discussion

Multiple neurohormonal changes generally cause hyponatremia in patients with ADHF, particularly renin-angiotensin aldosterone system (RAAS) activation to stimulate the release of arginine vasopressin. RAAS has an indirect impact on circulating the water-electrolyte balance through the autonomic nervous system and vasoactive peptides [18]. Creatinine and urea nitrogen concentrations are considered to be the renal function indicators [19], and their increase may indicate an abnormal renal function in the detected patients, leading to water-electrolyte imbalance under the disordered RAAS context. In our study, serum urea nitrogen level was detected to have a stronger association with hyponatremia in patients with ADHF. Similarly, existing research documented that a high serum urea nitrogen level was a risk factor for adverse ADHF events [20]. Serum urea nitrogen at a high level can increase hypoxia tolerance and lead to an increase in blood flow velocity per unit time concerning the mechanism of action; it can simultaneously aggravate the degree of atherosclerosis and eventually result in ADHF [21, 22]. Furthermore, cardiac dysfunction may increase brain natriuretic peptide secretion, which can act on the kidney, inhibit the renin and aldosterone release, increase urinary sodium excretion, and possibly lead to hyponatremia [23]. Meanwhile, diet is also an influential factor in the serum urea nitrogen level, especially in protein intake and endogenous protein catabolism [18, 24]. Patients with ADHF have increased consumption of energy and enhanced protein catabolism, further accelerating the discharge of urea nitrogen [25]. Therefore, it is important for medical workers to pay close attention to the patient’s renal function, especially the urea nitrogen level. There is a need to formulate detailed and personalised dietary plans to ensure the supplementation of high-quality protein and sodium [26].

Ikeda N et al. found in their research that there was a positive correlation between systolic blood pressure and blood sodium at admission, which was consistent with this study’s results [27]. The blood sodium level and extracellular volume in the general population both have an independent impact on the blood pressure level [28]; therefore, low blood sodium can induce hypotension. On the contrary, in the presence of hypotension, there is an activated RAAS system in vivo and long-term over-activation of the system, which may cause neuroendocrine mechanism disturbance and eventually lead to hyponatremia [29–31]. In this regard, ADHF patients’ sodium salt consumption should be controlled by the nursing staff, especially for patients with low systolic blood pressure [32]. Moreover, patients with cardiac function grades III and IV have more severe HF, accompanied by an increased degree of oedema, which may further aggravate hyponatremia occurrence. Hence, timely cardiac function correction and maintenance of systolic blood pressure stability are the keys to controlling subsequent disease deterioration.

In this study, the Nomogram prediction model was verified with an AUC of 0.757, and the calibration curve was near the ideal curve, indicating that the model has good discrimination. The prediction model for hyponatremia in patients with ADHF established in this study can intuitively and visually predict hyponatremia occurrence in ADHF patients.

However, our study has some limitations. Firstly, we only conducted a retrospective study in one hospital, so the sample population was limited, further limiting the use of nomograms. Secondly, some indicators were not included in the model because of the high rate of missing data, which decreased the comprehensiveness of the model. The accuracy of the model can be further improved if these missing variables, such as alanine aminotransferase, serum calcium ions and oedema, are added in future studies [33]. Thirdly, we only used the data for the internal verification of our model, and other external data still need to be used to further verify its performance and clinical applicability.

## Conclusion

The findings in our study suggest that there is a high hyponatremia risk in ADHF patients with a high creatinine level, a high serum urea nitrogen level, low systolic blood pressure, and cardiac function grades III and IV. This study establishes a risk prediction model for hyponatremia in patients with ADHF, which has good prediction ability. Hyponatremia risk screening in patients at the initial stage of admission can significantly benefit nursing staff to carry out scientific prevention and control, as well as precise implementation according to the specific risk factors.

## Data Availability

The data analyzed in this study are not publicly available due to the privacy policy of the hospital but are available from the corresponding author on reasonable request.
